# Team and Electronic Health Record Features and Burnout Among Family Physicians

**DOI:** 10.1001/jamanetworkopen.2024.42687

**Published:** 2024-11-05

**Authors:** Lisa S. Rotenstein, Nathaniel Hendrix, Robert L. Phillips, Julia Adler-Milstein

**Affiliations:** 1 Division of Clinical Informatics, Department of Medicine, University of California, San Francisco; 2 Division of General Internal Medicine, Department of Medicine, University of California, San Francisco; 3 Center for Physician Experience and Practice Excellence, Brigham and Women’s Hospital, Boston, Massachusetts; 4 The Center for Professionalism & Value in Health Care, American Board of Family Medicine, Washington, DC

## Abstract

**Importance:**

In the context of a growing volume of electronic health record (EHR)–based work and post–COVID-19 pandemic staffing pressures, health system leaders need an up-to-date understanding of changes in family physicians’ experiences of burnout, determinants of burnout, and how to enhance the family physicians’ experience.

**Objective:**

To evaluate the association of family physicians’ perceptions of team structure and EHR experiences with burnout and identify modifiable practice structure factors associated with team and EHR experiences.

**Design, Setting, and Participants:**

A serial cross-sectional survey study was conducted from December 1, 2016, to October 24, 2023. Participants included family physicians seeking continuous certification through the American Board of Family Medicine.

**Main Outcomes and Measures:**

Yearly prevalence of burnout, perceived team efficiency, perceived EHR proficiency, and perceived EHR time were the outcomes. Multivariable logistic regression models subsequently assessed associations of team- and EHR-related experiences with burnout and the association between practice structure and staffing features in team efficiency and EHR time.

**Results:**

The study included 10 315 physicians who answered the subset of questions related to burnout on the American Board of Family Medicine’s Continuous Certification Questionnaire between 2017 and 2023. Among the sample, 5584 respondents (54.1%) were male, and the median age was 50 (IQR, 43-58) years. The proportion of physicians reporting burnout ranged from 37.9% in 2017 to a peak of 42.8% in 2022, which did not represent a significant temporal trend (*P* = .91). Appropriate home EHR use was associated with 0.58 (95% CI, 0.53-0.64; *P* < .001) times the odds of burnout, while high team efficiency was associated with 0.61 (95% CI, 0.56-0.67; *P* < .001) times the odds of burnout. Physician collaboration with a registered nurse was associated with greater odds of high team efficiency (odds ratio [OR], 1.35; 95% CI, 1.22-1.50). Collaboration with a physician assistant was associated with greater odds of appropriate home EHR time (OR, 1.13; 95% CI, 1.03-1.24).

**Conclusions and Relevance:**

In this national cross-sectional study of US family physicians, appropriate time spent on the EHR at home and primary care team efficiency were associated with lower odds of burnout. These findings suggest that clinical leaders and policymakers should focus on optimizing primary care team support and family physicians’ EHR experiences to enhance the sustainability of primary care practice.

## Key Points

**Question:**

Are team structure and electronic health record (EHR) experiences associated with burnout among family physicians?

**Findings:**

In this cross-sectional study of 10 315 family physicians from 2017 to 2023, a continued substantial prevalence of burnout over time was observed, and perceived appropriate home EHR time and high team efficiency were associated with lower odds of burnout. Specific modifiable practice structures were associated with perceived high team efficiency and appropriate home EHR time.

**Meaning:**

The findings of this study suggest that clinical leaders and policymakers seeking to ensure sustainable primary care practice should prioritize adequate team supports and acceptable EHR workloads for family physicians.

## Teaser

This cross-sectional study examines the structure of the health care team and use of electronic health records in the context of the prevalence of burnout among physicians certified by the American Academy of Family Physicians.

## Introduction

Electronic health records (EHRs) are a key contributor to burnout,^1,2^ which affects nearly two-thirds of all physicians^3^ and half of family physicians.^4^ The COVID-19 pandemic has created a variety of new pressures for all physicians, including an increased volume of EHR-based work^5^ and pandemic-related staffing pressures.^6^ Across the physician workforce, this has resulted in burnout,^7^ intent to leave, and intent to reduce clinical hours.^8^ These issues are particularly salient for family physicians, who face the greatest burden of EHR time^9^ and who are in short supply across the US, particularly in rural areas.^10,11^

In this health care landscape, health system leaders need an up-to-date understanding of changes in family physicians experiences of burnout and how to enhance the family physicians’ experience to ensure the sustainability of this workforce. Burnout is a complex outcome and reducing it may be achieved by working on improving family physicians’ experiences in specific domains that are associated with burnout.^12^ For example, if leaders had evidence that family physicians’ positive perceptions of specific aspects of work were associated with lower burnout, and if actionable factors to improve these aspects of family physicians’ experiences were identified, health system leaders would then be equipped to implement targeted interventions and measure progress in addressing intermediate markers of physician burnout.

Prior work has identified that team culture^13^ and objective time spent on the EHR^1,2^ are associated with burnout. While single-site studies or studies with more narrow geographic or organizational focus have assessed how team environments^14,15^ and EHR experience perceptions^1^ are related to burnout, there is less evidence about these associations on a national scale that includes multiple practice setting types. Additionally, while prior work^15,16^ has suggested the importance of fully staffed, stable, and high-functioning teams for clinician experience, there is a critical evidence gap related to specific modifiable practice factors that influence positive perceptions of team structure and EHR experiences. These gaps limit the ability of operational leaders to undertake actionable changes that enhance family physicians’ EHR interactions, work environments, and ultimately, their experiences of care provision.

To address these gaps, we used data from the American Board of Family Medicine (ABFM) Continuous Certification Questionnaire (CCQ)^17^ to answer 3 main questions. How has the prevalence of burnout among family physicians changed since 2017? Which team structure and EHR experience perceptions are associated with burnout among family physicians? Which modifiable practice structure factors are associated with positive team structure and EHR experience perceptions?

## Methods

This research was determined to be exempt from human participants protections by the institutional review board at the American Academy of Family Physicians, with a waiver of informed consent due to use of secondary data. This study followed the Strengthening the Reporting of Observational Studies in Epidemiology (STROBE) reporting guideline.

### Study Population

The study population for this analysis was family physicians who completed the CCQ through the ABFM from December 1, 2016, to October 24, 2023. Family physicians seeking ABFM certification must complete the CCQ, and thus, this questionnaire has a 100% response rate. Certification takes place on a rolling 7- to 10-year basis, related to a move from a 7-year to a 10-year certification cycle.

### Survey Instrument

The core questions in the CCQ query respondents about their (1) demographic characteristics (sex, age, degree type), (2) clinical load, and (3) practice setting and resources (including practice location, size, and ownership). A question was added in 2022 that asks whether respondents’ primary practice site participates in 1 or more value-based care arrangements, such as a patient-centered medical home, accountable care organization, or pay-for-performance arrangement. Additional question modules are presented randomly to respondents to reduce overall question burden. One of these modules, which is the focus of our study and was presented to 20% of CCQ respondents, includes the following items, which are all derived from the Mini-Z^18^: perceived team efficiency, EHR time at home, EHR proficiency, and burnout (via a validated single-item measure).

### Sample Characteristics

Focusing on the survey respondents who were presented the survey module including Mini-Z questions, we first descriptively characterized the sample, including respondents’ degrees (MD vs DO), age (<35, 35-44, 45-54, 55-64, and ≥65 years), sex (male, female, or refused/other), location of practice (urban vs rural), size of practice site (solo practice, 2-5, 6-20, and >20 physicians), respondent’s practice ownership status (full owner of practice site, part owner, employee, contractor, refused/unknown), practice-site type (health system, independent, or other), and practice specialty mix (family medicine, primary care specialties, multiple specialties, blank). We described the prevalence of these characteristics across the sample of physicians both during the study period (2017-2013) and in each year.

### Primary Outcomes

#### Burnout Prevalence

We then quantified the prevalence of burnout by year and across the study period. High burnout was defined as an answer of once per week or more frequently to the question “I feel burned out from my work,” and low burnout was defined as an answer of “a few times a month” or less frequently.

#### Prevalence of Team Structure and EHR Experience Perceptions

We additionally quantified the prevalence of perceived team efficiency, perceived EHR proficiency, and perceived home EHR time by year and across the study period. High perceived team efficiency was defined as an answer of optimal or good to the question “The degree to which my care team works efficiently together is…” Answers of satisfactory, marginal, or poor to this question were defined as low perceived team efficiency. A high amount of time spent on the EHR was defined as an answer of moderately high or excessive to the statement “The amount of time I spend on the EHR at home is…”; answers of minimal/none, modest, or satisfactory were defined as appropriate home EHR time. High EHR proficiency was defined as answers of optimal or good to the statement “My proficiency with EHR use is…”; answers of satisfactory, marginal, or poor were considered low EHR proficiency.

### Secondary Outcomes

#### Association of Team Structure and EHR Experience Perceptions With Burnout

We subsequently built multivariable logistic regression models to assess the associations of team- and EHR-related experiences (team efficiency, home EHR time, and EHR proficiency) with burnout, controlling for physician age, degree, location, size of practice, employment status, and year. We then expanded on this base model by using interaction terms to assess how associations between team experiences and burnout and EHR-related experiences and burnout varied over time from 2017 to 2023.

#### Factors Associated With Team Structure and EHR Experience Perceptions

We built multivariable logistic regression models to assess whether practice structure and staffing features are associated with the team and EHR factors we identified as being related to burnout. We specifically assessed how practice location, practice site type, respondent’s practice ownership status, practice size, and respondent’s collaboration with specific team members (medical assistant, certified nursing assistant, licensed practical nurse, registered nurse, physician assistant, nurse practitioner, midwife, psychiatric nurse, psychiatrist, social worker, psychologist, physical or occupational therapist, pharmacist, or care coordinator) were associated with EHR and teamwork outcomes of interest. While our base models were for the years 2017 to 2023, since questions about participation in value-based care initiatives were only asked in 2023, we additionally developed models including only 2022 to 2023 data that assessed the association of the practice structure and staffing factors with our EHR and teamwork outcomes of interest.

### Statistical Analysis

All analyses were completed in R, version 4.2.2 (R Foundation for Statistical Computing). Wald χ^2^ tests on univariate linear regression were used to assess temporal trends in the prevalence of our primary outcomes of burnout, team efficiency, appropriate home EHR time, and high EHR proficiency. Wald χ^2^ tests were also used to assess adjusted odds ratios (ORs) in multivariable regression analyses with outcomes of burnout, high team efficiency, and appropriate home EHR time. A 2-sided α level of .05 was used to assess statistical significance.

## Results

### Sample Characteristics

The sample consisted of 10 315 physicians who answered the ABFM CCQ including the Mini-Z subset of questions from 2017 to 2023. As reported in the Table, the physicians were 88.8% MDs and 12.2% DOs. More than half (5584 [54.1%]) were male, 4690 (45.5%) were female, and the median age was 50 (IQR, 43-58) years. More than half (57.8%) of the respondents were employees, 11.3% were full owners of their practices, and 3.2% were contractors. While 10.0% of physicians in the sample practiced as solo physicians and 20.4% were in a practice with more than 20 physicians, the rest practiced in a setting with 2 to 19 physicians. More than three-fourths of the physicians practiced in an urban/suburban setting, and 13.5% practiced in a rural setting. Physicians were distributed across health systems (40.0%), academic centers (6.4%), independent practice (25.9%), and other settings (federally qualified health centers [3.8%], health maintenance organizations [6.1%]). Sample characteristics by year are displayed in eTable 1 in Supplement 1.

**Table.** Sample Characteristics

Characteristic	Survey year, 2017-2023 (N = 10 315)
Degree	
DO	1188 (11.5)
MD	9127 (88.5)
Age (categorical), y	
>35	151 (1.5)
35-44	2929 (28.4)
45-54	3654 (35.4)
55-64	2638 (25.6)
65 and over	943 (9.1)
Age, median (IQR), y	50 (43-58)
Sex	
Female	4690 (45.5)
Male	5584 (54.1)
Refused/other	41 (0.4)
Practice location	
Urban	7934 (76.9)
Rural	1392 (13.5)
Missing/blank	989 (9.6)
Site size	
Solo physician	1032 (10.0)
2-5 Physicians	2999 (29.1)
6-20 Physicians	2637 (25.6)
>20 Physicians	2108 (20.4)
Refused/unknown/blank	1539 (14.9)
Employment status	
Full owner	1170 (11.3)
Part owner	1309 (12.7)
Employee	5965 (57.8)
Contractor	332 (3.2)
Refused/unknown/blank	1539 (14.9)
Practice site type	
Health system	4125 (40.0)
Academic	663 (6.4)
Hospital/health system owned practice	2977 (28.9)
Managed care/HMO	485 (6.1)
Independent	2671 (25.9)
Other	3519 (34.1)
Federal	391 (3.8)
FQHC or look-alike	591 (5.7)
Government clinic, nonfederal	116 (1.1)
Indian health service	71 (0.7)
Rural health clinic (federally qualified)	201 (1.9)
Work site clinic	165 (1.6)
Other/unknown setting	1984 (19.2)
Practice specialty mix	
Family medicine only	3999 (38.8)
Primary care specialties only	2356 (22.8)
Multiple specialties	1788 (17.3)
Blank	2172 (21.1)

Abbreviations: FQHC, federally qualified health center; HMO, health maintenance organization.

### Burnout Prevalence

**Figure 1.**
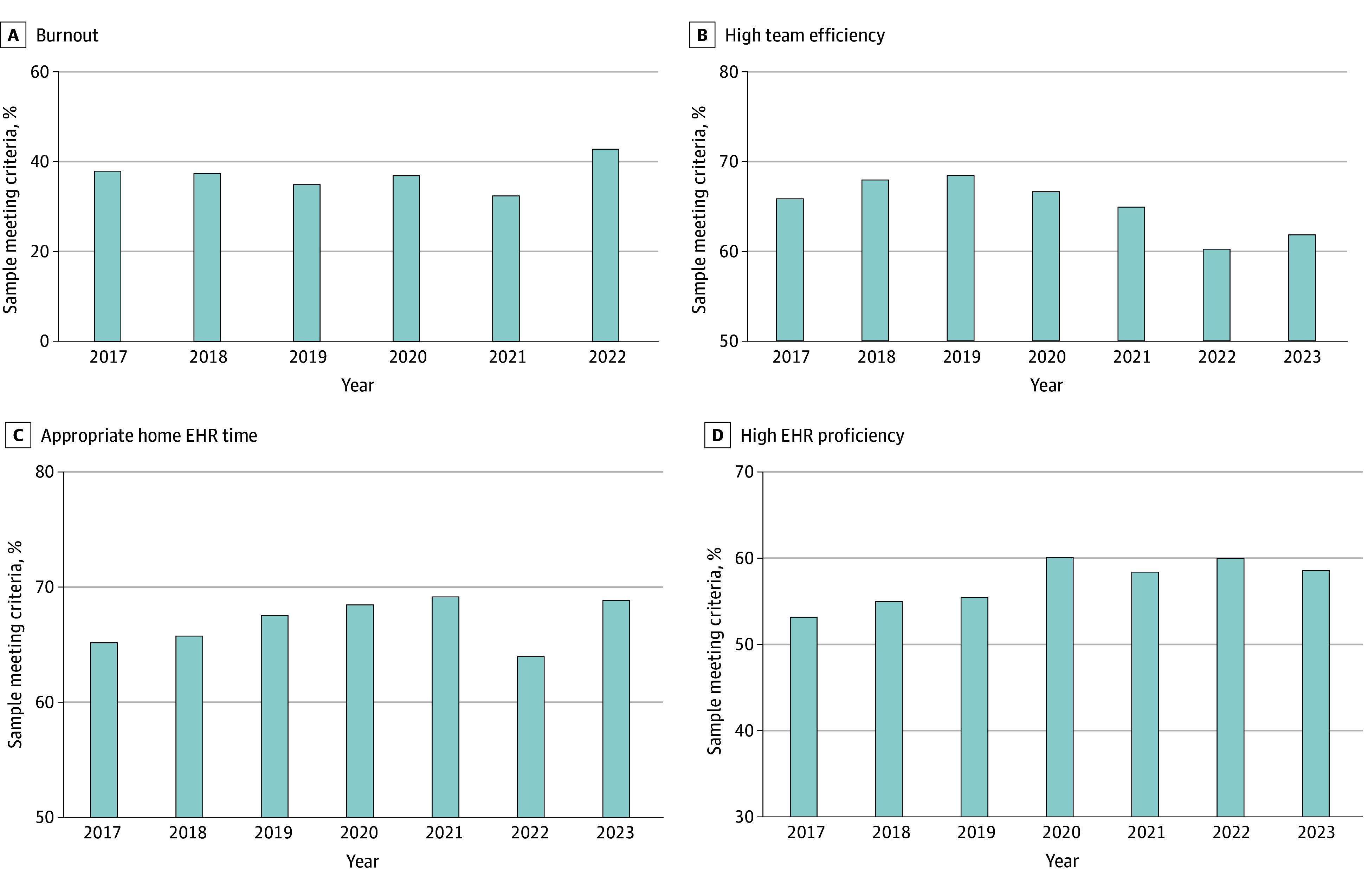
Prevalence of Burnout, Perceptions of Team Efficiency, Home Electronic Health Record (EHR) Time, and EHR Proficiency Among Family Physicians by Year

The proportion of physicians reporting burnout ranged from 37.9% in 2017 to 40.4% in 2023, with a peak of 42.8% in 2022 (Figure 1A). This did not represent a significant temporal trend (b = 0.005; *P* = .91).

### Prevalence of Team Structure and EHR Experience Perceptions

The prevalence of perceived team efficiency decreased over time, from 65.8% in 2017 to 61.8% in 2023, with a high of 68.4% in 2019 (b = −0.01; *P* = .04) (Figure 1B). The prevalence of appropriate home EHR time increased over the study period, with 53.1% of physicians reporting this outcome in 2017 vs 62.5% in 2020 and 58.5% in 2023 (b = 0.01; *P* = .03) (Figure 1C). The prevalence of high self-reported EHR proficiency remained stable over the study period (b = 0.002; *P* = .56), ranging from 65.2% in 2017 to 64.0% in 68.9% in 2023 (Figure 1D).

### Association of Team Structure and EHR Experience Perceptions With Burnout

**Figure 2.**
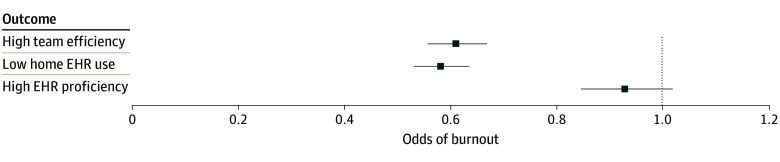
Cross-Sectional Adjusted Associations of Team Efficiency, Appropriate Home Electronic Health Record (EHR) Time, and High EHR Proficiency With Burnout Model was additionally adjusted for respondent age, medical degree type, sex, practice location, practice site type, respondent’s practice ownership status, practice size, practice specialty mix, and year.

Across the study period, appropriate home EHR use was associated with 0.58 (95% CI, 0.53-0.64; *P* < .001) times the odds of burnout, while high team efficiency was associated with an OR of 0.61 (95% CI, 0.56-0.67; *P* < .001) for burnout (Figure 2; eTable 2 in Supplement 1). When translated to a number needed to treat, these ORs suggest that 8 additional physicians perceiving appropriate home EHR time would result in prevention of 1 additional case of burnout, and 9 additional physicians perceiving high team efficiency would result in prevention of 1 case of burnout. Electronic health record proficiency was not associated with burnout (OR, 0.93; 95% CI, 0.85-1.02; *P* = .12). The association between high team efficiency and burnout did not change significantly over the study period (OR, 0.96; 95% CI, 0.92-1.00; *P* = .05) nor did the association between appropriate home EHR time and burnout (OR, 1.00; 95% CI, 0.97-1.04; *P* = .84).

### Association of Practice Structure Factors With Team Structure and EHR Experience Perceptions

**Figure 3.**
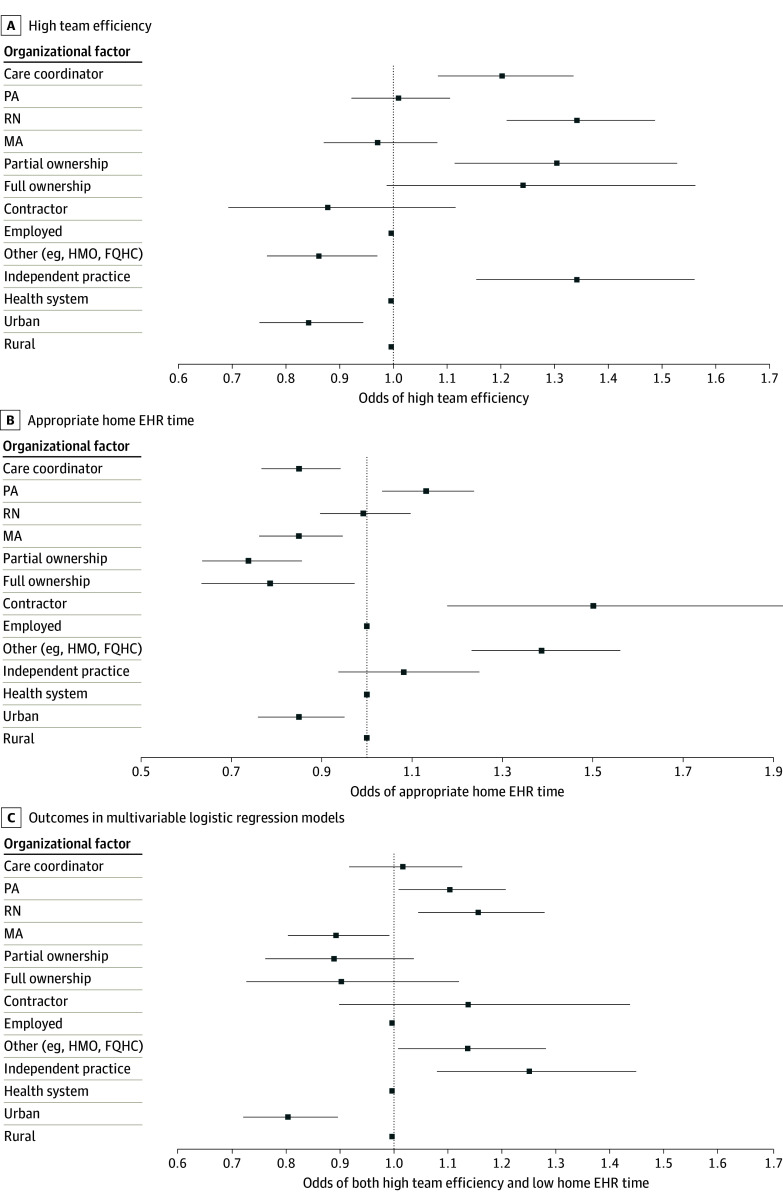
Organizational Factors Associated With High Team Efficiency, Appropriate Home Electronic Health Record (EHR) Time, and Both Outcomes in Multivariable Logistic Regression Models In addition to factors presented in the panels, models were additionally adjusted for respondent age, medical degree type, sex, practice specialty mix, and year. FQHC indicates federally qualified health center; HMO, health maintenance organization; MA, medical assistant; PA, physician assistant; RN, registered nurse.

In a model estimating high team efficiency (Figure 3A; eTable 3 in Supplement 1), the following factors were positively associated with this outcome: collaboration with a registered nurse (OR, 1.35; 95% CI, 1.22-1.50), collaboration with a care coordinator (OR, 1.21; 95% CI, 1.09-1.34), working for an independent practice vs a health system (OR, 1.35; 95% CI, 1.16-1.57), and partial practice ownership vs employment (OR, 1.31; 95% CI, 1.12-1.54). Full practice ownership vs employment was not associated with high team efficiency (OR, 1.25; 95% CI, 0.99-1.57). The following factors were associated with lower odds of high team efficiency: practicing in an urban compared with a rural setting (OR, 0.84; 95% CI, 0.75-0.95) and practicing for a non–health system nonindependent practice organization (eg, health maintenance organization, federally qualified health center vs independent practice) (OR, 0.86; 95% CI, 0.77-0.97). The number of physicians in the practice was not associated with perceptions of team efficiency.

As shown in Figure 3B and eTable 4 in Supplement 1, the following factors were associated with greater odds of appropriate home EHR time: collaboration with a physician assistant (OR, 1.13; 95% CI, 1.03-1.24) and practicing for a non–health system, nonindependent practice organization (eg, health maintenance organization, federally qualified health center vs independent practice) (OR, 1.39; 95% CI, 1.23-1.56). The following factors were associated with lower odds of appropriate home EHR time: practicing in an urban vs a rural setting (OR, 0.85; 95% CI, 0.76-0.95), full practice ownership vs employment (OR, 0.79; 95% CI, 0.63-0.97), partial practice ownership vs employment (OR, 0.74; 95% CI, 0.63-0.86), collaboration with a medical assistant (OR, 0.85; 95% CI, 0.76-0.95), and collaboration with a care coordinator (OR, 0.85; 95% CI, 0.77-0.94). Practicing in a clinic with 6 to 20 clinicians was associated with lower odds of appropriate home EHR time compared with solo practice (OR, 0.79; 95% CI, 0.64-0.98).

As shown in Figure 3C and eTable 5 in Supplement 1, the following factors were associated with greater odds of a joint outcome of high team efficiency and appropriate home EHR time: collaboration with a registered nurse (OR, 1.16; 95% CI, 1.05-1.29) and collaboration with a physician assistant (OR, 1.11; 95% CI, 1.01-1.21). Working in an urban setting was associated with lower odds of this outcome (OR, 0.81; 95% CI, 0.72-0.90), practicing in a clinic comprising more than 1 physician was associated with a lower odds of the joint outcome of high team efficiency and appropriate home EHR time for clinics with 2 to 5 clinicians (OR, 0.80; 95% CI, 0.66-0.98), 6 to 20 clinicians (OR, 0.73; 95% CI, 0.59-0.91), and more than 20 clinicians (OR, 0.77; 95% CI, 0.61-0.9).

**Figure 4.**
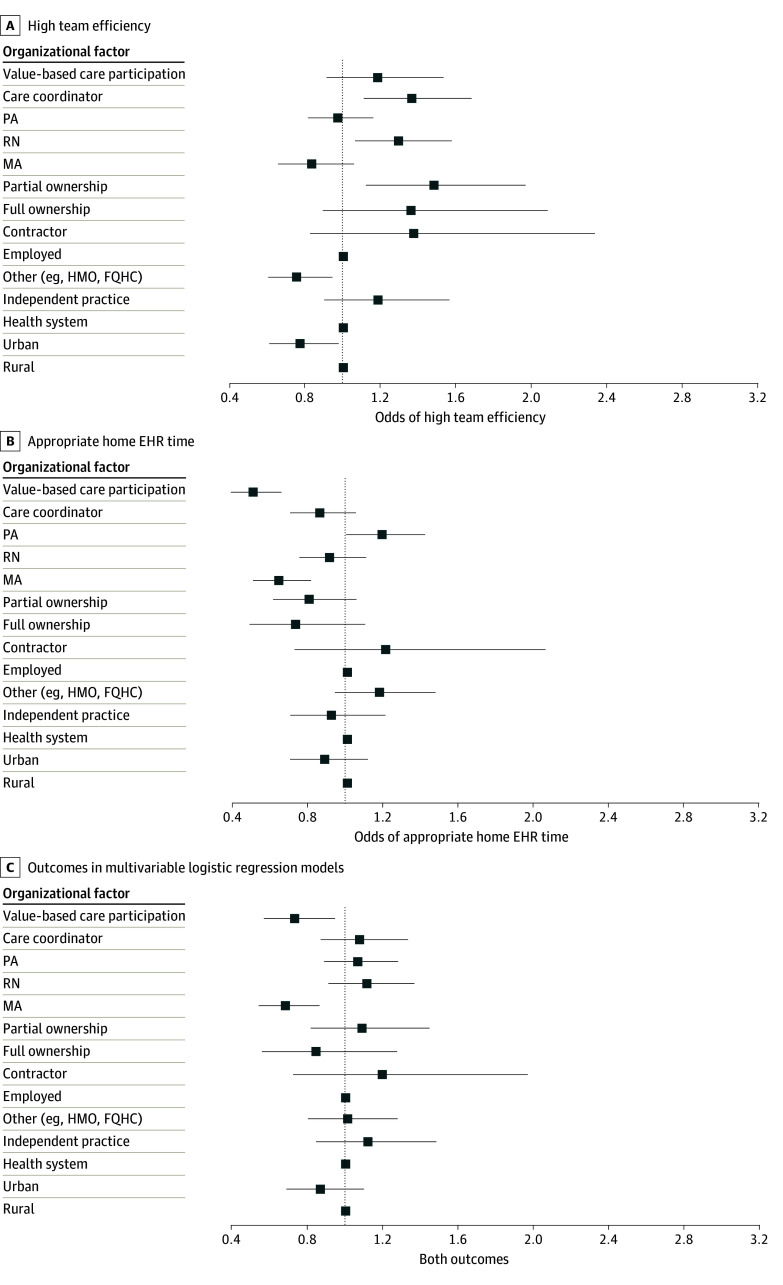
Organizational Factors Associated With High Team Efficiency, Appropriate Home Electronic Health Record (EHR) Time, and Both Outcomes in Multivariable Logistic Regression Models In addition to factors presented in the panels, models were additionally adjusted for respondent age, medical degree type, sex, practice specialty mix, and year. Models were based on 2022-2023 data alone since these were the only years when a question about value-based care participation was asked in the survey. FQHC indicates federally qualified health center; HMO, health maintenance organization; MA, medical assistant; PA, physician assistant; RN, registered nurse.

In models based on 2022 to 2023 data only (Figure 4A-C; eTables 6-8 in Supplement 1), value-based care participation was associated with lower odds of appropriate home EHR time (OR, 0.5; 95% CI, 0.4-0.7) and of the joint outcome of high team efficiency and appropriate home EHR time (OR, 0.7; 95% CI, 0.6-0.9). Value-based care participation was not associated with high team efficiency.

## Discussion

In this national serial cross-sectional study with a 100% yearly response rate, we observed a continued substantial prevalence of burnout among US family physicians, ranging from 37.9% in 2017 to 40.4% in 2023 after a peak of 42.8% in 2022. We found that high team efficiency and appropriate home EHR time are associated with lower odds of burnout among family physicians, with stable findings from 2017 to 2023. Compared with standard preventive interventions, such as statin prescription,^19^ the number needed to treat to avoid a case of burnout implied by the respective ORs for appropriate home EHR time and high team efficiency suggests that optimizing these aspects of physician experience may be useful for addressing physician burnout. Specific practice staffing features, such as collaborating with a registered nurse, were associated with greater odds of high team efficiency and appropriate home EHR time. These results underscore a need for clinical leaders to enhance the capacity and functioning of primary care teams while continuing efforts to minimize home EHR time. Both these goals appear to be potentiated by specific practice structure and staffing features.

Our findings regarding the association of appropriate home EHR time and high team efficiency with lower odds of burnout are consistent with prior evidence in this domain. Among many examples,^20^ in a study of family physicians at an academic medical center, clinicians in the top quartiles of time spent on the EHR after hours on scheduled clinic days had greater odds of emotional exhaustion.^2^ Across Rhode Island, physicians reporting moderately high or excessive time on the EHR at home had nearly 3 times the odds of emotional exhaustion compared with those with no or minimal EHR use.^21^ An extensive primary care literature has reported the crucial role of effective teams in influencing burnout. For example, in a study of more than 4500 primary care personnel from 588 Veteran’s Administration Clinics, participation in a fully staffed patient-aligned care team, a staffing unit comprising a primary care clinician, nurse care manager, administrative clerk, and licensed practical nurse or medical assistant, was associated with lower burnout.^16^ Having a fully staffed team was associated with lower burnout, while high turnover, which can impede team efficiency, was associated with greater odds of burnout.^15^ Ensuring team efficiency and stability is a particularly salient issue at a time when many members of the health care workforce report high levels of work overload and intent to leave the job^6^ and given the substantial prevalence of burnout observed in ours’ and others’ studies.^3^

We additionally observed the association of family physicians’ collaboration with specific staff professions with perceptions of team efficiency and low EHR time. Specifically, collaboration with a nurse was associated with greater odds of team efficiency, while working with a physician assistant was associated with greater odds of low EHR time. Collaboration with a care coordinator was associated with higher odds of perceiving high team efficiency. While those collaborating with a care coordinator were less likely to perceive appropriate home EHR time, it is possible that this is due to practices employing care coordinators having more complex patient populations, which we were unable to control for in this survey-based study. Overall, the results of our analysis provide actionable evidence for health care leaders pursuing staffing-focused approaches to primary care physicians’ perceptions of teamwork, manageable EHR workload, and ultimately, the overall experience of care delivery. The staffing-focused approaches informed by our analyses likely complement individual physician-focused approaches previously described in the literature to optimize EHR time, such as individual EHR training and tailored EHR coaching,^22,23^ as well as approaches focused on the EHR, such as EHR optimization sprints.^24,25^ Ultimately, a combination of team, individual, and EHR interface-focused approaches will likely be needed to make progress on primary care physicians’ work experiences.

It is notable that practice ownership was associated with greater odds of high team efficiency but lower odds of appropriate home EHR time in our analysis. Multiple studies have suggested positive associations of practice ownership compared with employed practice with burnout, ability to improve quality of care, and EHR satisfaction.^13,26,27^ Compared with employed practice, participating in the ownership structure of a practice may allow clinicians to have greater control over team structure and functioning, likely leading to enhanced perceptions of team efficiency and/or greater agency for modifying the practice team. However, to the extent that physicians with an ownership stake in their practice more directly feel pressures related to staffing, finances, and meeting documentation requirements for reimbursement, this may result in greater after-hours work, including on the EHR. These tradeoffs between enhanced control over the practice environment but potentially greater responsibilities and demands for nonemployed physicians are important to consider as the proportion of physician employment continues to grow.^28^

Our finding of physicians in urban compared with rural practices having lower adjusted odds of high team efficiency, appropriate home EHR time, or both outcomes is more surprising. While prior evidence observed greater implementation of patient-centered medical home components in rural compared with urban primary care clinics, potentially due to clinic organizational factors,^29^ other studies have reported lesser adoption of EHR systems among family medicine practitioners in rural settings^30^ and a lower likelihood of primary care practitioners having EHRs with certain advanced functionalities in rural settings.^31^ Future studies should explore in more depth the reasons behind the urban and rural differences identified in this study.

The subset analysis using 2022 to 2023 data noted that value-based care participation was associated with lower odds of appropriate EHR time and lower odds of the joint outcome of high team efficiency and appropriate EHR time. These results suggest that the documentation or clinical care pressures associated with value-based care arrangements may ultimately contribute to greater home EHR burden, with potential downstream implications for physician experience. While some have suggested the potential ability of value-based care programs to benefit the physician experience by enhancing physician time with patients,^32^ our results highlight the potentially conflicting influence of value-based care arrangements on physicians’ work and experience. This is consistent with prior evidence reporting, for example, that participation in an accountable care organization was associated with increased burnout among primary care physicians in small- and medium-sized practices nationwide.^33^ Potential causes include a greater focus on and documentation burden from quality measurement and reporting in value-based payment models and a lack of consistent translation of value-based payment programs into the additional staffing needed to care for and document a range of patient needs.

### Strengths and Limitations 

Our study has both strengths and limitations. The strengths include the large sample size of more than 10 000 physicians and more than 40% of board-certified family physicians across 6 years, a 100% response rate to the survey that serves as the basis of the study, and consistency of findings over time.

Regarding limitations, while survey respondents shared their perceptions of their EHR time and EHR proficiency, these factors were not objectively measured. It is possible that respondents’ perceptions do not perfectly align with their true EHR time expenditure or proficiency. Additionally, while we present longitudinal estimates of our outcomes of interest, specific respondents were not followed up over time, and thus our longitudinal estimates are based on serial, cross-sectional samples.

## Conclusions

The findings of this study suggest that time spent on the EHR at home and primary care team efficiency is associated with primary care physician burnout, with associations also persisting from 2017 to 2023. Potentially modifiable practice structure factors related to team composition and reduction of EHR burden are associated with a greater likelihood of these outcomes. These findings have particular importance given a continued substantial prevalence of burnout among family physicians. Clinical leaders and policymakers seeking to develop care delivery models that enable sustainable primary care practice should focus on ensuring adequate team support and acceptable EHR workloads for physicians.

## Notes

Supplement 1. **eTable 1.** Characteristics of Study Sample by Year **eTable 2.** Multivariable Model Assessing Associations of Team Efficiency, Appropriate Home EHR Time, and High EHR Proficiency With Burnout **eTable 3.** Multivariable Model Assessing Organizational Factors Associated With High Team Efficiency **eTable 4.** Multivariable Model Assessing Organizational Factors Associated With Appropriate Home EHR Time **eTable 5.** Multivariable Model Assessing Organizational Factors Associated With Both High Team Efficiency and Appropriate Home EHR Time **eTable 6.** Multivariable Model Assessing Organizational Factors Associated With High Team Efficiency, Including Value-Based Care Participation **eTable 7.** Multivariable Model Assessing Organizational Factors Associated With Appropriate Home EHR Time, Including Value-Based Care Participation **eTable 8.** Multivariable Model Assessing Organizational Factors Associated With High Team Efficiency and Appropriate Home EHR Time, Including Value-Based Care Participation

Supplement 2.
Data Sharing Statement
