# Supplementary material for: Team and Electronic Health Record Features and Burnout Among Family Physicians
Source: JAMA Netw Open. 2024 Nov 5;7(11):e2442687. doi: 10.1001/jamanetworkopen.2024.42687 (PMC11539011; doi:10.1001/jamanetworkopen.2024.42687)
Supplement: Supplement 1. — eTable 1. Characteristics of Study Sample by Year eTable 2. Multivariable Model Assessing Associations of Team Efficiency, Appropriate Home EHR Time, and High EHR Proficiency With Burnout eTable 3. Multivariable Model Assessing Organizational Factors Associated With High Team Efficiency eTable 4. Multivariable Model Assessing Organizational Factors Associated With Appropriate Home EHR Time eTable 5. Multivariable Model Assessing Organizational Factors Associated With Both High Team Efficiency and Appropriate Home EHR Time eTable 6. Multivariable Model Assessing Organizational Factors Associated With High Team Efficiency, Including Value-Based Care Participation eTable 7. Multivariable Model Assessing Organizational Factors Associated With Appropriate Home EHR Time, Including Value-Based Care Participation eTable 8. Multivariable Model Assessing Organizational Factors Associated With High Team Efficiency and Appropriate Home EHR Time, Including Value-Based Care Participation [file jamanetwopen-e2442687-s001.pdf]

Supplementary Online Content

Rotenstein S, Hendrix N, Phillips RL, Adler-Milstein J. Team and electronic health record features and burnout among family physicians. *JAMA Netw Open*. 2024;7(11):e2442687. doi:10.1001/jamanetworkopen.2024.42687

eTable 1. Characteristics of Study Sample by Year
eTable 2. Multivariable Model Assessing Associations of Team Efficiency, Appropriate Home EHR Time, and High EHR Proficiency With Burnout
eTable 3. Multivariable Model Assessing Organizational Factors Associated With High Team Efficiency
eTable 4. Multivariable Model Assessing Organizational Factors Associated With Appropriate Home EHR Time
eTable 5. Multivariable Model Assessing Organizational Factors Associated With Both High Team Efficiency and Appropriate Home EHR Time
eTable 6. Multivariable Model Assessing Organizational Factors Associated With High Team Efficiency, Including Value-Based Care Participation
eTable 7. Multivariable Model Assessing Organizational Factors Associated With Appropriate Home EHR Time, Including Value-Based Care Participation
eTable 8. Multivariable Model Assessing Organizational Factors Associated With High Team Efficiency and Appropriate Home EHR Time, Including Value-Based Care Participation

This supplementary material has been provided by the authors to give readers additional information about their work.

© 2024 Rotenstein S et al. *JAMA Network Open.*

eTable 1. Characteristics of Study Sample by Year

		2017	2018	2019	2020	2021	2022	2023
**Total respondents (N)**		**1901**	**1747**	**1686**	**762**	**928**	**1105**	**2186**
**Degree = MD (%)**		1701 (89.5)	1567 (89.7)	1530 (90.7)	640 (84.0)	795 (85.7)	956 (86.5)	1938 (88.7)
**Age (categorical)**	**Under 35**	24 (1.3)	17 (1.0)	21 (1.2)	36 (4.7)	28 (3.0)	14 (1.3)	11 (0.5)
	**35-44**	424 (22.3)	381 (21.8)	384 (22.8)	365 (47.9)	422 (45.5)	468 (42.4)	485 (22.2)
	**45-54**	778 (40.9)	709 (40.6)	646 (38.3)	213 (28.0)	253 (27.3)	343 (31.0)	712 (32.6)
	**55-64**	547 (28.8)	480 (27.5)	484 (28.7)	111 (14.6)	161 (17.3)	190 (17.2)	665 (30.4)
	**65+**	128 (6.7)	160 (9.2)	151 (9.0)	37 (4.9)	64 (6.9)	90 (8.1)	313 (14.3)
**Age, median (IQR)**		51 (45, 58)	51 (45, 59)	52 (45, 59)	44 (40, 53)	45 (40, 54)	47 (41, 56)	53 (46, 61)
**Sex**	**Female**	817 (43.0)	742 (42.5)	752 (44.6)	399 (52.4)	460 (49.6)	550 (49.8)	970 (44.4)
	**Male**	1084 (57.0)	1005 (57.5)	934 (55.4)	363 (47.6)	467 (50.3)	555 (50.2)	1176 (53.8)
	**Other / Refused**	0 (0.0)	0 (0.0)	0 (0.0)	0 (0.0)	1 (0.1)	0 (0.0)	40 (1.8)
**Metropolitan status**	**Urban**	1416 (74.5)	1254 (71.8)	1302 (77.2)	566 (74.3)	666 (71.8)	866 (78.4)	1864 (85.3)
	**Rural**	271 (14.3)	238 (13.6)	242 (14.4)	95 (12.5)	117 (12.6)	141 (12.8)	288 (13.2)
	**Missing / Blank**	214 (11.3)	255 (14.6)	142 (8.4)	101 (13.3)	145 (15.6)	98 (8.9)	34 (1.6)
**Site size**	**Solo practice**	218 (11.5)	206 (11.8)	167 (9.9)	59 (7.7)	86 (9.3)	97 (8.8)	199 (9.1)
	**2-5 Providers**	575 (30.2)	496 (28.4)	496 (29.4)	236 (31.0)	258 (27.8)	291 (26.3)	647 (29.6)
	**6-20 Providers**	497 (26.1)	450 (25.8)	431 (25.6)	185 (24.3)	241 (26.0)	279 (25.2)	554 (25.3)
	**>20 Providers**	332 (17.5)	344 (19.7)	346 (20.5)	165 (21.7)	190 (20.5)	265 (24.0)	466 (21.3)
	**Refused / Unknown / Blank**	279 (14.7)	251 (14.4)	246 (14.6)	117 (15.4)	153 (16.5)	173 (15.7)	320 (14.6)
**Site owner**	**Full owner**	242 (12.7)	216 (12.4)	203 (12.0)	75 (9.8)	92 (9.9)	114 (10.3)	228 (10.4)
	**Part owner**	271 (14.3)	237 (13.6)	225 (13.3)	77 (10.1)	104 (11.2)	139 (12.6)	256 (11.7)
	**Employee**	1055 (55.5)	980 (56.1)	964 (57.2)	472 (61.9)	543 (58.5)	640 (57.9)	1311 (60.0)
	**Contractor**	54 (2.8)	63 (3.6)	48 (2.8)	21 (2.8)	36 (3.9)	39 (3.5)	71 (3.2)

© 2024 Rotenstein S et al. *JAMA Network Open.*

	**Refused / Unknown / Blank**	279 (14.7)	251 (14.4)	246 (14.6)	117 (15.4)	153 (16.5)	173 (15.7)	320 (14.6)
**Practice site type**	**Health system**	717 (37.7)	682 (39.0)	683 (40.5)	332 (43.6)	367 (39.5)	445 (40.3)	899 (41.1)
	**Academic**	116 (6.1)	99 (5.7)	104 (6.2)	63 (8.3)	57 (6.1)	83 (7.5)	141 (6.5)
	**Hospital / health system owned practice**	520 (27.4)	500 (28.6)	500 (29.7)	229 (30.1)	264 (28.4)	308 (27.9)	656 (30.0)
	**Managed care / HMO practice**	81 (4.3)	83 (4.8)	79 (4.7)	40 (5.2)	46 (5.0)	54 (4.9)	102 (4.7)
	**Independent**	567 (29.8)	487 (27.9)	453 (26.9)	167 (21.9)	203 (21.9)	266 (24.1)	528 (24.2)
	**Other**	617 (32.5)	578 (33.1)	550 (32.6)	263 (34.5)	358 (38.6)	394 (35.7)	759 (34.7)
	**Federal**	67 (3.5)	69 (3.9)	63 (3.7)	30 (3.9)	37 (4.0)	38 (3.4)	87 (4.0)
	**FQHC or look-alike**	88 (4.6)	97 (5.6)	84 (5.0)	48 (6.3)	71 (7.7)	61 (5.5)	142 (6.5)
	**Gov't clinic, Non-Federal**	22 (1.2)	23 (1.3)	19 (1.1)	10 (1.3)	7 (0.8)	11 (1.0)	24 (1.1)
	**Indian Health Service**	15 (0.8)	10 (0.6)	13 (0.8)	5 (0.7)	8 (0.9)	11 (1.0)	9 (0.4)
	**Rural Health Clinic (federally qualified)**	43 (2.3)	30 (1.7)	26 (1.5)	16 (2.1)	19 (2.0)	27 (2.4)	40 (1.8)
	**Work site clinic**	26 (1.4)	22 (1.3)	28 (1.7)	8 (1.0)	19 (2.0)	22 (2.0)	40 (1.8)
	**Other / Unknown**	356 (18.7)	327 (18.7)	317 (18.8)	137 (18.0)	197 (21.2)	224 (20.3)	417 (19.1)
**Practice specialty mix**	**Family medicine only**	787 (41.1)	709 (40.6)	669 (39.7)	279 (36.6)	327 (35.2)	378 (34.2)	850 (38.9)
	**Primary care specialties only**	412 (21.7)	349 (20.0)	375 (22.2)	194 (25.5)	220 (23.7)	289 (26.2)	517 (23.7)
	**Multiple specialties**	311 (16.4)	321 (18.4)	290 (17.2)	122 (16.0)	172 (18.5)	213 (19.3)	359 (16.4)
	**Blank**	391 (20.6)	368 (21.1)	352 (20.9)	167 (21.9)	209 (22.5)	225 (20.4)	460 (21.0)

© 2024 Rotenstein S et al. *JAMA Network Open.*

eTable 2. Multivariable Model Assessing Associations of Team Efficiency, Appropriate Home EHR Time, and High EHR Proficiency with Burnout

	B	Wald X^2 Value	SE	Odds Ratio	Lower CI (2.5%)	Upper CI (97.5%)
High EHR Proficiency	-0.07	0.117	0.05	0.93	0.85	1.02
High EHR Home Use	0.54	<0.001	0.05	1.72	1.57	1.88
Low Team Efficiency	0.49	<0.001	0.05	1.64	1.49	1.79
Year	0.01	0.376	0.01	1.01	0.99	1.03
MD Degree (vs. DO)	0.04	0.549	0.07	1.04	0.91	1.20
Age Category (vs. Age < 35)						
Age 35-44	0.40	0.051	0.20	1.49	1.01	2.24
Age 45-44	0.31	0.122	0.20	1.37	0.93	2.07
Age 55-65	0.24	0.233	0.20	1.28	0.86	1.93
Age 65+	-0.28	0.200	0.22	0.76	0.50	1.17
Sex (vs. Female)						
Male	-0.14	0.003	0.05	0.87	0.79	0.95
Other	-0.06	0.849	0.31	0.94	0.51	1.69
Urban Setting (vs. Rural)	-0.04	0.557	0.06	0.97	0.86	1.09
Size of Practice Site (vs. solo practices)						
2-5 Clinicians	0.35	0.001	0.12	1.42	1.15	1.77
6-20 Clinicians	0.38	0.001	0.11	1.46	1.16	1.84
> 20 Clinicians	0.27	0.033	0.12	1.30	1.02	1.66
Site Ownership (vs. Employed)						
Contractor	-0.32	0.023	0.14	0.72	0.55	0.95
Full Ownership	0.04	0.755	0.12	1.04	0.82	1.31
Partial Ownership	0.08	0.310	0.08	1.09	0.93	1.27
Main Practice Site Structure (vs. Health System)						
Independent	-0.06	0.475	0.08	0.95	0.81	1.10
Other (HMO, FQHC)	-0.07	0.259	0.06	0.93	0.83	1.05
Specialty Composition of Site (vs. Family Medicine only)						
Multiple Specialties	-0.04	0.528	0.07	0.96	0.84	1.10
Primary Care Specialty Mix	-0.10	0.099	0.06	0.91	0.81	1.02

© 2024 Rotenstein S et al. *JAMA Network Open.*

eTable 3. Multivariable Model Assessing Organizational Factors Associated with High Team Efficiency

	B	Wald X^2 Value	SE	Odds Ratio	Lower CI (2.5%)	Upper CI (97.5%)
Year	-0.04	<0.001	0.01	0.96	0.95	0.98
MD Degree (vs. DO)	0.00	0.967	0.07	1.00	0.87	1.14
Age Category (vs. Age < 35)						
Age 35-44	-0.37	0.048	0.18	0.69	0.48	0.99
Age 45-44	-0.37	0.044	0.18	0.69	0.48	0.98
Age 55-65	-0.28	0.135	0.19	0.76	0.52	1.08
Age 65+	-0.10	0.598	0.20	0.90	0.61	1.32
Sex (vs. Female)						
Male	0.26	<0.001	0.04	1.29	1.18	1.41
Other	-0.80	0.005	0.29	0.45	0.25	0.78
Urban Setting (vs. Rural)	-0.17	0.004	0.06	0.84	0.75	0.95
Size of Practice Site (vs. solo practices				0.76	0.50	1.17
2-5 Clinicians	-0.03	0.802	0.11	0.82	0.65	1.05
6-20 Clinicians	-0.13	0.269	0.12	0.97	0.79	1.20
>20 Clinicians	-0.19	0.116	0.12	0.88	0.70	1.11
Site Ownership (vs. Employed)						
Contractor	-0.13	0.299	0.12	0.88	0.69	1.12
Full Ownership	0.22	0.059	0.12	1.25	0.99	1.57
Partial Ownership	0.27	0.001	0.08	1.31	1.12	1.54
Main Practice Site Structure (vs. Health System)						
Independent	0.30	<0.001	0.08	1.35	1.16	1.57
Other (HMO, FQHC)	-0.15	0.017	0.06	0.86	0.77	0.97
Specialty Composition of Site (vs. Family Medicine only)				-	-	-
Multiple Specialties	0.05	0.426	0.07	1.06	0.92	1.20
Primary Care Specialty Mix	-0.12	0.039	0.06	0.89	0.80	0.99
Collaboration with a Medical Assistant	-0.03	0.642	0.06	0.97	0.87	1.09
Collaboration with a Certified Nursing Assistant	0.02	0.760	0.06	1.02	0.90	1.15
Collaboration with a Licensed Practical Nurse	-0.05	0.334	0.05	0.96	0.87	1.05
Collaboration with an RN	0.30	<0.001	0.05	1.35	1.22	1.50
Collaboration with a Physician Assistant	0.01	0.768	0.05	1.01	0.93	1.11
Collaboration with a Nurse Practitioner	-0.03	0.474	0.05	0.97	0.88	1.06
Collaboration with a Midwife	0.00	0.982	0.11	1.00	0.81	1.23
Collaboration with a Psychiatric Nurse	-0.10	0.289	0.10	0.91	0.76	1.09
Collaboration with a Psychiatrist	-0.16	0.028	0.07	0.85	0.74	0.98
Collaboration with a Social Worker	-0.02	0.715	0.06	0.98	0.87	1.10
Collaboration with a Psychologist	0.06	0.320	0.06	1.06	0.94	1.20
Collaboration with a PharmD	0.02	0.738	0.06	1.02	0.91	1.15
Collaboration with a Care Coordinator	0.19	<0.001	0.05	1.21	1.09	1.34
Collaboration with No Other Team Members	-0.21	0.087	0.13	0.81	0.63	1.03

© 2024 Rotenstein S et al. *JAMA Network Open.*

eTable 4. Multivariable Model Assessing Organizational Factors Associated with Appropriate Home EHR Time

	B	Wald X^2 Value	SE	Odds Ratio	Lower CI (2.5%)	Upper CI (97.5%)
Year	0.06	<0.001	0.01	1.06	1.04	1.08
MD Degree (vs. DO)	0.00	0.977	0.07	1.00	0.88	1.14
Age Category (vs. Age < 35)						
Age 35-44	-0.63	0.001	0.19	0.53	0.36	0.78
Age 45-44	-0.89	<0.001	0.19	0.41	0.28	0.59
Age 55-65	-0.98	<0.001	0.20	0.38	0.25	0.55
Age 65+	-0.97	<0.001	0.20	0.38	0.25	0.56
Sex (vs. Female)						
Male	0.40	<0.001	0.04	1.48	1.36	1.62
Other	-0.18	0.529	0.29	0.83	0.47	1.47
Urban Setting (vs. Rural)	-0.16	0.005	0.06	0.85	0.76	0.95
Size of Practice Site (vs. Solo practice)						
2-5 Clinicians	-0.20	0.046	0.10	0.82	0.67	1
6-20 Clinicians	-0.23	0.035	0.11	0.79	0.64	0.98
> 20 Clinicians	-0.14	0.238	0.12	0.87	0.69	1.1
Site Ownership (vs. Employed)						
Contractor	0.41	0.001	0.12	1.50	1.18	1.92
Full Ownership	-0.24	0.027	0.11	0.79	0.63	0.97
Partial Ownership	-0.30	<0.001	0.08	0.74	0.63	0.86
Main Practice Site Structure (vs. Health System)						
Independent	0.08	0.285	0.07	1.08	0.94	1.25
Other (HMO, FQHC)	0.33	<0.001	0.06	1.39	1.23	1.56
Specialty Composition of Site (vs. Family Medicine only)						
Multiple Specialties	0.09	0.174	0.06	1.09	0.96	1.24
Primary Care Specialty Mix	0.00	0.962	0.05	1.00	0.90	1.12
Collaboration with a Medical Assistant	-0.16	0.003	0.06	0.85	0.76	0.95
Collaboration with a Certified Nursing Assistant	0.15	0.012	0.06	1.16	1.03	1.31
Collaboration with a Licensed Practical Nurse	-0.07	0.119	0.05	0.93	0.85	1.02
Collaboration with an RN	-0.01	0.875	0.05	0.99	0.90	1.10
Collaboration with a Physician Assistant	0.12	0.007	0.05	1.13	1.03	1.24
Collaboration with a Nurse Practitioner	-0.12	0.010	0.05	0.88	0.81	0.97
Collaboration with a Midwife	-0.17	0.114	0.11	0.85	0.69	1.04
Collaboration with a Psychiatric Nurse	0.08	0.407	0.09	1.08	0.90	1.29
Collaboration with a Psychiatrist	0.07	0.365	0.07	1.07	0.93	1.23
Collaboration with a Social Worker	-0.10	0.080	0.06	0.90	0.80	1.01
Collaboration with a Psychologist	0.02	0.788	0.06	1.02	0.90	1.14
Collaboration with a PharmD	0.03	0.598	0.06	1.03	0.92	1.16
Collaboration with a Care Coordinator	-0.16	0.002	0.05	0.85	0.77	0.94
Collaboration with No Other Team Members	-0.23	0.060	0.12	0.79	0.62	1.01

© 2024 Rotenstein S et al. *JAMA Network Open.*

eTable 5. Multivariable Model Assessing Organizational Factors Associated with Both High Team Efficiency and Appropriate Home EHR Time

	B	Wald X^2 Value	SE	Odds Ratio	Lower CI (2.5%)	Upper CI (97.5%)
Year	0.03	0.005	0.01	1.03	1.01	1.05
MD Degree (vs. DO)	0.04	0.588	0.07	1.04	0.91	1.18
Age Category (vs. Age <35)						
Age 35-44	-0.46	0.008	0.17	0.63	0.45	0.89
Age 45-44	-0.66	<0.001	0.17	0.52	0.37	0.73
Age 55-65	-0.64	<0.001	0.17	0.53	0.37	0.74
Age 65+	-0.51	0.006	0.18	0.60	0.42	0.86
Sex (vs. Female)						
Male	0.35	<0.001	0.04	1.42	1.30	1.54
Other	-0.68	0.043	0.34	0.51	0.25	0.95
Urban Setting (vs. Rural)	-0.22	<0.001	0.06	0.81	0.72	0.90
Size of Practice Site (vs. Solo practice)						
2-5 Clinicians	-0.22	0.029	0.10	0.80	0.66	0.98
6-20 Clinicians	-0.31	0.006	0.11	0.73	0.59	0.91
> 20 Clinicians	-0.27	0.023	0.12	0.77	0.61	0.96
Site Ownership (vs. Employed)						
Contractor	0.13	0.269	0.12	1.14	0.9	1.45
Full Ownership	-0.10	0.370	0.11	0.9	0.73	1.13
Partial Ownership	-0.11	0.147	0.08	0.89	0.76	1.04
Main Practice Site Structure (vs. Health System)						
Independent	0.23	0.002	0.08	1.26	1.08	1.46
Other (HMO, FQHC)	0.13	0.031	0.06	1.14	1.01	1.29
Specialty Composition of Site (vs. Family Medicine only)				1.14	1.00	1.29
Multiple Specialties	0.13	0.055	0.07	0.95	0.85	1.06
Primary Care Specialty Mix	-0.05	0.341	0.06	-	-	-
Collaboration with a Medical Assistant	-0.11	0.040	0.05	0.90	0.81	1.00
Collaboration with a Certified Nursing Assistant	0.08	0.192	0.06	1.08	0.96	1.21
Collaboration with a Licensed Practical Nurse	-0.07	0.107	0.05	0.93	0.85	1.02
Collaboration with an RN	0.15	0.004	0.05	1.16	1.05	1.29
Collaboration with a Physician Assistant	0.10	0.026	0.05	1.11	1.01	1.21
Collaboration with a Nurse Practitioner	-0.11	0.023	0.05	0.90	0.82	0.99
Collaboration with a Midwife	-0.12	0.287	0.11	0.89	0.72	1.10
Collaboration with a Psychiatric Nurse	-0.02	0.849	0.09	0.98	0.82	1.17
Collaboration with a Psychiatrist	-0.03	0.688	0.07	0.97	0.84	1.12
Collaboration with a Social Worker	-0.05	0.424	0.06	0.95	0.85	1.07
Collaboration with a Psychologist	0.03	0.628	0.06	1.03	0.91	1.16
Collaboration with a PharmD	0.03	0.643	0.06	1.03	0.92	1.15
Collaboration with a Care Coordinator	0.02	0.706	0.05	0.85	0.77	0.94
Collaboration with No Other Team Members	-0.23	0.061	0.12	0.79	0.62	1.01

© 2024 Rotenstein S et al. *JAMA Network Open.*

eTable 6. Multivariable Model Assessing Organizational Factors Associated with High Team Efficiency, Including Value-Based Care Participation

	B	Wald X^2 Value	SE	Odds Ratio	Lower CI (2.5%)	Upper CI (97.5%)
Value-Based Care Participation	0.17	0.208	0.13	1.18	0.91	1.53
MD Degree (vs. DO)	-0.03	0.798	0.13	0.97	0.75	1.24
Age Category (vs. Age < 35)						
Age 35-44	-0.54	0.241	0.46	0.58	0.22	1.41
Age 45-44	-0.43	0.358	0.46	0.65	0.25	1.59
Age 55-65	-0.36	0.444	0.47	0.7	0.27	1.71
Age 65+	-0.10	0.842	0.48	0.91	0.34	2.28
Sex (vs. Female)						
Male	0.33	<0.001	0.09	1.39	1.18	1.65
Other	-0.69	0.060	0.37	0.5	0.24	1.02
Urban Setting (vs. Rural)	-0.26	0.031	0.12	0.77	0.61	0.98
Size of Practice Site						
2-5 Clinicians	-0.03	0.895	0.24	0.97	0.65	1.46
6-20 Clinicians	-0.11	0.638	0.21	0.9	0.58	1.4
> 20 Clinicians	-0.34	0.146	0.23	0.71	0.44	1.13
Site Ownership (vs. Employed)						
Contractor	0.32	0.230	0.26	1.37	0.82	2.33
Full Ownership	0.31	0.157	0.22	1.36	0.89	2.08
Partial Ownership	0.39	0.006	0.14	1.48	1.12	1.97
Main Practice Site Structure (vs. Health System)						
Independent	0.17	0.231	0.14	1.18	0.9	1.56
Other (HMO, FQHC)	-0.28	0.013	0.11	0.75	0.6	0.94
Specialty Composition of Site (vs. Family Medicine only)						
Multiple Specialties	0.09	0.441	0.12	1.1	0.87	1.4
Primary Care Specialty Mix	-0.14	0.172	0.10	0.87	0.72	1.06
Collaboration with a Medical Assistant	-0.18	0.133	0.12	0.83	0.65	1.06
Collaboration with a Certified Nursing Assistant	0.01	0.911	0.12	1.01	0.8	1.3
Collaboration with a Licensed Practical Nurse	-0.22	0.015	0.09	0.8	0.67	0.96
Collaboration with an RN	0.26	0.011	0.10	1.29	1.06	1.58
Collaboration with a Physician Assistant	-0.03	0.741	0.09	0.97	0.81	1.16
Collaboration with a Nurse Practitioner	0.04	0.671	0.09	1.04	0.87	1.25
Collaboration with a Midwife	0.04	0.861	0.21	1.04	0.69	1.56
Collaboration with a Psychiatric Nurse	0.10	0.577	0.17	1.1	0.78	1.55
Collaboration with a Psychiatrist	-0.14	0.350	0.15	0.87	0.65	1.17
Collaboration with a Social Worker	-0.02	0.853	0.12	0.98	0.78	1.23
Collaboration with a Psychologist	0.06	0.582	0.12	1.07	0.85	1.35
Collaboration with a PharmD	0.16	0.163	0.11	1.17	0.94	1.46
Collaboration with a Care Coordinator	0.31	0.004	0.11	1.36	1.11	1.68
Collaboration with No Other Team Members	-0.02	0.957	0.30	0.98	0.55	1.79

© 2024 Rotenstein S et al. *JAMA Network Open.*

eTable 7. Multivariable Model Assessing Organizational Factors Associated With Appropriate Home EHR Time, Including Value-Based Care Participation

	B	Wald X^2 Value	SE	Odds Ratio	Lower CI (2.5%)	Upper CI (97.5%)
Value-Based Care Participation	-0.70	<0.001	0.14	0.5	0.38	0.65
MD Degree (vs. DO)	0.21	0.100	0.13	1.23	0.96	1.58
Age Category (vs. Age < 35)						
Age 35-44	-1.00	0.059	0.53	0.37	0.12	0.97
Age 45-44	-1.30	0.014	0.53	0.27	0.09	0.72
Age 55-65	-1.46	0.006	0.53	0.23	0.07	0.62
Age 65+	-1.34	0.013	0.54	0.26	0.08	0.71
Sex (vs. Female)						
Male	0.54	<0.001	0.09	1.72	1.46	2.04
Other	-0.45	0.240	0.38	0.64	0.29	1.33
Urban Setting (vs. Rural)	-0.13	0.275	0.12	0.88	0.69	1.11
Size of Practice Site (vs. Solo practice)						
2-5 Clinicians	-0.29	0.149	0.20	0.75	0.5	1.11
6-20 Clinicians	-0.50	0.026	0.22	0.61	0.39	0.94
> 20 Clinicians	-0.48	0.041	0.24	0.62	0.39	0.98
Site Ownership (vs. Employed)						
Contractor	0.18	0.489	0.27	1.2	0.72	2.06
Full Ownership	-0.32	0.125	0.21	0.72	0.48	1.09
Partial Ownership	-0.23	0.104	0.14	0.8	0.6	1.05
Main Practice Site Structure (vs. Health System)						
Independent	-0.09	0.520	0.14	0.91	0.7	1.2
Other (HMO, FQHC)	0.16	0.173	0.12	1.17	0.93	1.47
Specialty Composition of Site (vs. Family Medicine only)				-	-	-
Multiple Specialties	0.07	0.570	0.12	1.07	0.84	1.36
Primary Care Specialty Mix	0.02	0.864	0.10	1.02	0.84	1.23
Collaboration with a Medical Assistant	-0.45	<0.001	0.12	0.63	0.5	0.81
Collaboration with a Certified Nursing Assistant	0.27	0.032	0.12	1.3	1.02	1.66
Collaboration with a Licensed Practical Nurse	-0.21	0.021	0.09	0.81	0.68	0.97
Collaboration with an RN	-0.10	0.323	0.10	0.9	0.74	1.1
Collaboration with a Physician Assistant	0.17	0.060	0.09	1.18	0.99	1.41
Collaboration with a Nurse Practitioner	-0.14	0.139	0.09	0.87	0.73	1.05
Collaboration with a Midwife	-0.14	0.497	0.21	0.87	0.58	1.3
Collaboration with a Psychiatric Nurse	0.27	0.123	0.18	1.31	0.93	1.85
Collaboration with a Psychiatrist	-0.03	0.865	0.15	0.97	0.72	1.31
Collaboration with a Social Worker	-0.06	0.607	0.12	0.94	0.75	1.18
Collaboration with a Psychologist	0.10	0.376	0.12	1.11	0.88	1.4
Collaboration with a PharmD	-0.02	0.835	0.11	0.98	0.78	1.22
Collaboration with a Care Coordinator	-0.16	0.125	0.10	0.85	0.69	1.05
Collaboration with No Other Team Members	-0.16	0.597	0.30	0.85	0.48	1.55

© 2024 Rotenstein S et al. *JAMA Network Open.*

eTable 8. Multivariable Model Assessing Organizational Factors Associated with High Team Efficiency and Appropriate Home EHR Time, Including Value-Based Care Participation

	B	Wald X^2 Value	SE	Odds Ratio	Lower CI (2.5%)	Upper CI (97.5%)
Value-Based Care Participation	-0.32	0.016	0.13	0.73	0.57	0.94
MD Degree (vs. DO)	0.20	0.128	0.13	1.23	0.95	1.6
Age Category (vs. Age < 35)						
Age 35-44	-0.87	0.053	0.45	0.42	0.17	1.02
Age 45-44	0.96	0.033	0.45	0.38	0.16	0.94
Age 55-65	-1.01	0.026	0.45	0.36	0.15	0.9
Age 65+	-0.81	0.079	0.46	0.44	0.18	1.11
Sex (vs. Female)						
Male	0.45	<0.001	0.09	1.61	1.36	1.92
Other	-1.13	0.039	0.54	0.32	0.09	0.85
Urban Setting (vs. Rural)	-0.14	0.230	0.12	0.87	0.69	1.1
Size of Practice Site (vs. Solo practice)						
2-5 Clinicians	-0.41	0.039	0.20	0.66	0.45	0.98
6-20 Clinicians	-0.66	0.003	0.22	0.52	0.33	0.8
> 20 Clinicians	-0.74	0.002	0.23	0.48	0.3	0.75
Site Ownership (vs. Employed)						
Contractor	0.18	0.485	0.25	1.19	0.72	1.97
Full Ownership	-0.17	0.417	0.21	0.84	0.56	1.27
Partial Ownership	0.08	0.571	0.15	1.09	0.81	1.45
Main Practice Site Structure (vs. Health System)						
Independent	0.11	0.437	0.14	1.12	0.84	1.48
Other (HMO, FQHC)	0.01	0.930	0.12	1.01	0.8	1.28
Specialty Composition of Site (vs. Family Medicine only)				-	-	-
Multiple Specialties	0.15	0.248	0.13	1.16	0.9	1.48
Primary Care Specialty Mix	0.00	0.993	0.10	1	0.82	1.22
Collaboration with a Medical Assistant	-0.38	0.001	0.12	0.68	0.54	0.86
Collaboration with a Certified Nursing Assistant	0.13	0.311	0.13	1.14	0.89	1.45
Collaboration with a Licensed Practical Nurse	-0.28	0.003	0.09	0.76	0.63	0.91
Collaboration with an RN	0.11	0.304	0.10	1.11	0.91	1.36
Collaboration with a Physician Assistant	0.06	0.507	0.09	1.06	0.89	1.28
Collaboration with a Nurse Practitioner	-0.05	0.617	0.10	0.95	0.79	1.15
Collaboration with a Midwife	-0.07	0.761	0.22	0.94	0.6	1.43
Collaboration with a Psychiatric Nurse	0.17	0.329	0.18	1.19	0.84	1.68
Collaboration with a Psychiatrist	0.18	0.239	0.16	1.2	0.88	1.63
Collaboration with a Social Worker	-0.05	0.700	0.12	0.95	0.75	1.21
Collaboration with a Psychologist	0.03	0.777	0.12	1.04	0.81	1.32
Collaboration with a PharmD	0.02	0.870	0.12	1.02	0.81	1.28
Collaboration with a Care Coordinator	0.07	0.513	0.11	1.07	0.87	1.33
Collaboration with No Other Team Members	-0.25	0.389	0.29	0.78	0.44	1.37

© 2024 Rotenstein S et al. *JAMA Network Open.*
