# Supplementary material for: Team and Electronic Health Record Features and Burnout Among Family Physicians
Source: JAMA Netw Open. 2024 Nov 5;7(11):e2442687. doi: 10.1001/jamanetworkopen.2024.42687 (PMC11539011; doi:10.1001/jamanetworkopen.2024.42687)
Supplement: Supplement 2. — Data Sharing Statement [file jamanetwopen-e2442687-s002.pdf]

Data Sharing Statement

Rotenstein. Team and Electronic Health Record Features and Burnout Among Family Physicians. *JAMA Netw Open*. Published November 04, 2024.
doi:10.1001/jamanetworkopen.2024.42687

Data
Data available: No

Additional Information
Explanation for why data not available: Proprietary ABFM data
